# Cholinesterase inhibitors and reduced risk of hospitalization and mortality in patients with Alzheimer's dementia and heart failure

**DOI:** 10.1093/ehjcvp/pvae091

**Published:** 2025-01-07

**Authors:** Marianne Reimers-Wessberg, Hong Xu, Johan Fastbom, Åke Seiger, Maria Eriksdotter

**Affiliations:** Division of Clinical Geriatrics, Department of Neurobiology, Care Sciences and Society, Karolinska Institutet, Alfred Nobels allé 8, 171 77 Stockholm, Sweden; Research and Development Unit, Stockholms Sjukhem, 112 19 Stockholm, Sweden; Division of Clinical Geriatrics, Department of Neurobiology, Care Sciences and Society, Karolinska Institutet, Alfred Nobels allé 8, 171 77 Stockholm, Sweden; Aging Research Center, Department of Neurobiology, Care Sciences and Society, Karolinska Institutet, 171 65 Stockholm, Sweden; Division of Clinical Geriatrics, Department of Neurobiology, Care Sciences and Society, Karolinska Institutet, Alfred Nobels allé 8, 171 77 Stockholm, Sweden; Division of Clinical Geriatrics, Department of Neurobiology, Care Sciences and Society, Karolinska Institutet, Alfred Nobels allé 8, 171 77 Stockholm, Sweden; Theme Inflammation and Aging, Department of Aging, Karolinska University Hospital, 141 86 Stockholm, Sweden

**Keywords:** Heart failure, Alzheimer's disease, Cholinesterase inhibitors, Hospitalization, Mortality

## Abstract

**Aims:**

Cholinesterase inhibitors (ChEIs) have beneficial effects on the heart. Associations between ChEI-use and reduced mortality and cardiovascular events in Alzheimer's disease (AD) have been shown. Whether these associations exist in those with both heart failure (HF) and AD is unknown.

**Methods and results:**

A propensity score (PS) matched cohort with patients with HF and AD was obtained through linking registers for cognitive/dementia disorders, comorbidities, drug prescription, and death, in Sweden, to analyse associations between ChEI-use and risk of mortality or hospitalization for HF, stroke, or myocardial infarction, were examined. In 455 patients with and 455 without ChEI treatment, ChEI use was associated with reductions of mortality and hospitalization due to HF by 21% [0.79; (confidence interval) CI 0.66–0.96] and 47% (0.53; CI 0.38–0.75), respectively. Donepezil and galantamine but not rivastigmine were associated with a lower risk of death compared with non-users. Donepezil was associated with a lower risk of hospitalization due to HF compared with non-users. There was no significant difference in hospitalization for bradycardia, AV block, or implantation of pacemaker between ChEI use and non-use.

**Conclusion:**

This study suggests that in persons with HF and AD, treatment with ChEIs is associated with improved survival and a decreased risk of hospital care for HF, but results due to the type of ChEI vary.

## Introduction

The improved treatment during the last decades of diseases such as cancer and cardiovascular disorders have increased the incidences of chronic diseases as Alzheimer's dementia (AD)^1^ and heart failure (HF).^2^

Treatment of vascular risk factors has been associated with a lower incidence of dementia^3^ and a slower decline in AD.^4^

Changes in the autonomic nervous system^5^ (ANS) among patients with AD^6,7^ and with HF^6^ have been demonstrated. The parasympathetic activation has been shown to decrease during the development of AD, along with a more dominant role of sympathetic activation, leading to an increase in cardiovascular disorders.^6^ Also, deficits in central cholinergic function observed in AD could lead to autonomic dysfunction.^7^ These alterations in the ANS actively contribute to cardiac disease progression.

Inflammation plays a distinct role in AD and has also been suggested to play a key role in cardiac diseases, especially in HF. Murphy^8^ reported that in HF the strongest associations of inflammatory markers are found in HF with preserved ejection fraction (HFpEF), the most common form of HF in the elderly.

Acetylcholine (ACh) has been shown to have anti-inflammatory properties and the role of cholinergic signalling may be a key regulator of cardiac inflammation.^9^ Khuanjing *et al.*^10^ have in a review article demonstrated improved autonomic and cardiac functions of cholinesterase inhibitors (ChEIs) through various mechanisms including direct action of ACh on anti-arrhythmogenic, anti-apoptotic, anti-oxidative, anti-inflammatory, anti-hypertrophic, and anti-fibrotic processes. In fact, the main cardiovascular effects such as reduction of heart rate, and improved contractility and haemodynamic conditions^11^ could actively contribute to the positive effects on the heart in treatment with various drugs with heart rate-reducing effects, including beneficial effects on the inflammasome in cardiovascular disease. Inflammasomes, being large intracellular multiprotein complexes, promote inflammatory molecules as cytokines and interleukins with direct effects not only on atherosclerosis but also on HF.^12^

Still, in elderly patients there may be a fear of negative chronotropic effects when treating patients with ChEIs due to changes in regulatory functions in the vascular system, but it has been reported that none of the three ChEIs were associated with increased negative chronotropic, arrhythmogenic, and hypotensive effects for the elderly patients with AD.^13^ Isik *et al.*^14^ also reported that galantamine was not associated with significantly altered ECG parameters or arterial blood pressure in these elderly patients with AD, and further that donepezil^15^ or rivastigmine^16^ were also not associated with increased negative chronotropic, arrhythmogenic, or hypotensive effects among AD patients’.

Moreover, amyloid beta (Aβ) has been found to accumulate in the heart of patients with AD^17^ and accumulation of Aβ40 in blood has been associated with cardiac dysfunction and cardiovascular mortality.^18^

ChEIs are approved treatments in AD. The ChEIs reversibly, irreversibly, or pseudo-reversibly act by blocking acetylcholineesterase enzymes (AChE) and butyrylcholinesterase (BuChE) from breaking down ACh, which results in increased ACh levels in the synaptic cleft.^19^ ChEIs have been shown to be associated with modest cognitive benefits persisting over long-term^20^ as well as, by our group and others, a longer life expectancy.^21,6,20,22^

Using national epidemiology data, we have shown that in AD patients, ChEI use is associated with reduced risk for myocardial infarction,^21^ stroke,^23^ and death.^20,21,23,24^ Hsiao^25^ reported significantly fewer cardiovascular events and a reduction of cardiovascular death among AD patients treated with ChEI compared with non-users. It has further been shown that donepezil and rivastigmine could reduce HF hospitalization^26^ and that donepezil could reduce natriuretic peptide levels.^27^

Evidence of a possible risk reduction for cardiovascular events or mortality by ChEIs in persons with both cardiovascular disease and AD is scarce.

One of the most common cardiovascular disorders is HF. In a Swedish dementia population of 27 000 persons, the prevalence of HF was 15%.^28^ ChEIs have well known pulse reducing effects^29^ and although the risk for bradycardia must be considered, reducing heart rate is an important target for treatment of HF.

Thus, ChEIs may have beneficial effects on HF via both cardiac innervation and inflammation. However, there is no data on the ChEIs and risk for new HF events and mortality in an AD-population with HF. The aim of this study was therefore to investigate whether treatment with ChEI among persons with HF prior to the AD diagnosis, would be associated with decreased risk for hospitalization due to cardiovascular events or mortality and to investigate whether differences exist between the different ChEIs.

## Methods

### Study population

**Figure 1:**
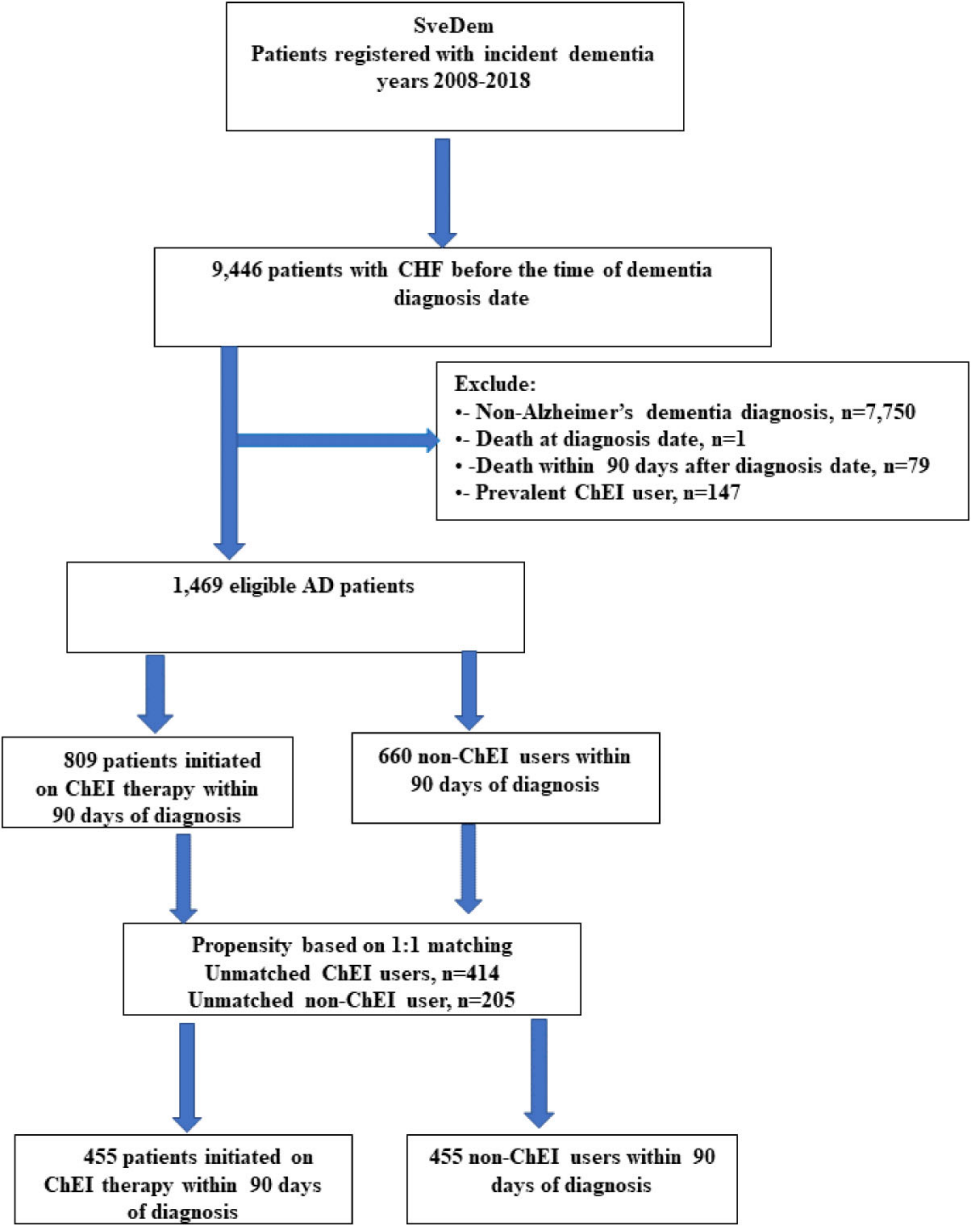
Flowchart of two groups with or without treatment with Cholinesterase inhibitors for comparisons.

This study was based on the Swedish registry of cognitive/dementia disorders; SveDem, www.svedem.se. SveDem is a web-based registry established in 2007 with the aim to register all incident dementia patients in Sweden with annual follow-up. The baseline registration in SveDem is initiated at the time of the dementia diagnosis. For this study, SveDem was record-linked with the National Patient Registry to obtain diagnoses of comorbidities, made in specialist clinics and hospitals, the Prescribed Drug Registry to obtain data on prescribed medications, the Total Population Registry, and the Causes of Death Registry to obtain death dates. We identified 9446 patients registered in SveDem with a dementia diagnosis between 1 January 2008, and 16 October 2018, who had at least one hospitalization with a diagnosis of HF [International Classification of Diseases (ICD)-10 scores I099, I110, I130, I132, I255, I420, I425-429, I43, I50, P290, Supplementary material online, *Table S1*] prior to the dementia diagnosis. Exclusion criteria were patients who had non-AD dementia (*n* = 7750), were dead at the diagnosis date (*n* = 1) or within 3 months thereafter (*n* = 79) and patients treated with ChEI before the dementia diagnosis date (*n* = 147). Of 1469 eligible AD patients, 809 were initiated with ChEI within 90 days, and 660 were not (non-ChEI users, *Figure 1*).

### Cholinesterase inhibitor exposure

The studied exposure was initiation of ChEIs therapy with donepezil, galantamine, or rivastigmine within 3 months of the dementia diagnosis, vs. no initiation within 3 months. Our primary analysis used an intention-to-treat design and assumed study exposures to be constant until end of follow up. The study index date was set 3 months after the dementia diagnosis date.

### Covariates

Study covariates were defined at the date of the study entry (from the diagnosis date and up to 3 months after the dementia diagnosis): age, sex, comorbidities (based on the ICD-10 codes): alcohol abuse, atrial fibrillation, cerebrovascular disease, chronic kidney disease (CKD), chronic pulmonary disease, depression, diabetes, fractures, hearing loss, hypertension, liver disease, myocardial infarction, obesity, peptic ulcer disease, peripheral vascular disease, rheumatic disease, and stroke; and medications in the ATC groups: angiotensin-converting enzyme inhibitors (ACEIs), angiotensin receptor blockers (ARBs), acetylsalicylic acid, antipsychotics, antidepressants, antithrombotics, anxiolytics, beta-blockers, calcium channel blockers, diuretics, aldosterone antagonists, hypnotics, memantine, nonsteroidal anti-inflammatory drugs and statins. For ICD-10 codes and ATC codes please see Supplementary material online, *Table S1*.

### Outcomes

The study outcome was all-cause death confirmed from the Cause of death registry and hospitalization due to composite, or separate events, of HF, stroke, or myocardial infarction (MI) from 3 months after the dementia diagnosis date to death or to the end of the follow-up on 16 October 2018, whichever occurred first, see Supplementary material online, *Table S2*. We also reported any hospitalization for ChEIsʹ known severe side effects of bradycardia, atrioventricular (AV) block, or need for pacemaker implantation. Patients were followed from the dementia diagnosis date until death, or the end of follow up in 16 October 2018, whichever occurred first. For ICD codes for outcome, please see Supplementary material online, *Table S2*.

### Data analyses

We performed 1:1 propensity score (PS) matching (without replacement) using the nearest-neighbor matching method and with a caliper of 0.01 to balance confounders between patients who used ChEIs and those who did not. We estimated the PS for ChEI using logistic regression models based on age, sex, diagnosis at the memory clinic, living situation (alone or in a nursing home), patient comorbidities, and current medications. The balance of baseline characteristics before and after matching is shown in *Tables 1* and *2*.

**Table 1:** Baseline characteristics, comorbidities and medication of the total HF-AD cohort stratified by ChEI treatment status within 3 months after diagnosis prior to propensity score matching

Baseline characteristics	HF and AD non-ChEI user *n* = 660	HF and AD ChEI-user *n* = 809	*P*-value
AD diagnosis	100.0%	100.0%	
Age, mean (SD)	84.2 (6.3)	81.5 (6.5)	<0.001***
Age strata			<0.001***
<70	2.7%	5.3%	
70–79	17.3%	28.3%	
80–89	60.3%	58.7%	
≥90	19.7%	7.7%	
Female	60.3%	57.0%	0.20
MMSE baseline, mean (SD)	19.4 (5.3)	21.5 (4.3)	<0.001***
MMSE strata			<0.001***
0–9	4.7%	0.7%	
10–19	38.9%	27.6%	
20–24	35.6%	44.0%	
≥25	15.3%	25.8%	
MMSE not recorded/done	5.5%	1.9%	
Diagnosed in specialist/memory clinic	58.0%	56.5%	0.55
Living alone	49.4%	47.7%	0.52
Living in nursing home	14.8%	6.3%	<0.001***
Comorbidities			
CCI, mean (SD)	4.0 (1.9)	3.9 (1.8)	0.17
Alcohol abuse	2.0%	1.9%	0.87
Atrial fibrillation	57.6%	51.7%	0.024*
Cerebrovascular diseases	18.6%	16.1%	0.19
Chronic kidney disease	12.6%	6.7%	<0.001***
Chronic pulmonary disease	19.5%	20.1%	0.77
Depression	9.7%	7.9%	0.23
Diabetes	25.3%	26.2%	0.69
Fractures	35.0%	28.6%	0.008**
Hearing loss	14.5%	12.5%	0.25
Hypertension	70.0%	69.5%	0.83
Liver disease	1.7%	1.1%	0.36
Myocardial Infarction	31.8%	31.6%	0.94
Peptic ulcers disease	7.0%	5.7%	0.31
Peripheral vascular disease	9.1%	12.2%	0.054
Rheumatic diseases	8.6%	6.7%	0.16
Stroke	11.7%	8.3%	0.030*
Medication			
ACEI/ARB	67.3%	73.5%	0.009**
Acetylsalicylic acid	47.4%	50.2%	0.29
Antipsychotics	8.0%	4.6%	0.006**
Antidepressants	30.8%	29.3%	0.54
Antithrombotics	90.8%	90.9%	0.95
Anxiolytics	24.2%	17.9%	0.003**
Beta-blocker	74.5%	75.3%	0.75
Calcium channel blocker	21.5%	23.9%	0.29
ChEI use			<0.001***
Non	100.0%	0.0%	
Donepezil	0.0%	61.1%	
Rivastigmine	0.0%	20.5%	
Galantamine	0.0%	18.4%	
Diuretics	73.6%	69.1%	0.056
Aldosterone antagonist	17.4%	20.1%	0.18
Hypnotics	32.1%	29.9%	0.36
Memantine	39,8%	5,2%	<0.001***
NSAID	6.2%	7.9%	0.21
Statins	38.5%	50.4%	<0.001***

MMSE, mini-mental state examination; ACEI, angiotensin-converting enzyme inhibitors; ARB, angiotensin receptor blockers; NSAIDs, nonsteroidal anti-inflammatory drugs.

*P < 0.05, **P < 0.01, ***P < 0.001.

**Table 2:** Baseline characteristics, comorbidities and medication stratified by ChEI treatment within 3 months in the propensity score matched cohort

Baseline characteristics	HF and AD Non-ChEI user *n* = 455	HF and AD ChEI-user *n* = 455	*P*-value
Age, mean (SD)	83.2 (6.2)	83.2 (5.7)	0.92
Age strata			0.71
<70	2.9%	3.3%	
70–79	20.7%	18.2%	
80–89	64.2%	67.3%	
≥90	12.3%	11.2%	
Female	58.5%	58.2%	0.95
MMSE baseline, mean (SD)	20.5 (4.9)	20.5 (4.4)	0.80
MMSE strata			0.44
0–9	2.4%	1.1%	
10–19	33.4%	35.6%	
20–24	41.3%	43.1%	
≥25	20.4%	17.6%	
MMSE not recorded/not done	2.4%	2.6%	
Diagnosed in specialist/memory clinic	57.8%	57.1%	0.84
Living alone	49.9%	50.8%	0.79
Nursing home	10.1%	9.2%	0.65
**Comorbidities**			
CCI, mean (SD)	3.9 (1.9)	3.9 (1.9)	0.71
Alcohol abuse	2.0%	1.8%	0.81
Atrial fibrillation	55.8%	57.1%	0.69
Cerebrovascular diseases	16.5%	18.0%	0.54
Chronic kidney disease	7.7%	8.8%	0.55
Chronic pulmonary disease	21.3%	20.7%	0.81
Depression	9.2%	10.1%	0.65
Diabetes	24.6%	24.0%	0.82
Fractures	30.8%	29.2%	0.61
Hearing loss	13.8%	13.2%	0.77
Hypertension	70.0%	69.5%	0.85
Liver disease	1.8%	1.3%	0.59
Myocardial infarction	31.0%	31.9%	0.78
Peptic ulcer	6.2%	6.4%	0.89
Peripheral vascular disease	10.3%	9.7%	0.74
Rheumatic diseases	7.9%	7.7%	0.90
Stroke	9.7%	10.1%	0.82
**Medication**			
ACEI/ARB	69.5%	69.7%	0.94
Acetylsalicylic acid	48.1%	49.7%	0.64
Antidepressants	30.3%	28.4%	0.51
Antipsychotics	6.8%	6.2%	0.69
Antithrombotic	91.2%	91.6%	0.81
Anxiolytics	22.2%	23.5%	0.64
Beta-blocker	72.5%	73.8%	0.65
Calcium channel blocker	22.6%	22.6%	1.00
ChEI use			<0.001***
Non	100.0%	0.0%	
Donepezil	0.0%	63.5%	
Rivastigmine	0.0%	19.3%	
Galantamine	0.0%	17.1%	
Diuretics	70.5%	71.2%	0.83
Aldosterone antagonist	18.9%	18.5%	0.86
Hypnotics	31.6%	32.3%	0.83
Memantine	42.4%	5.9%	<0.001***
NSAID	7.3%	6.8%	0.80
Statins	43.1%	40.7%	0.46

MMSE, mini-mental state examination; ACEI, angiotensin-converting enzyme inhibitors; ARB, angiotensin receptor blockers; NSAIDs, nonsteroidal anti-inflammatory drugs.

***P < 0.001.

Continuous variables are presented as means with standard deviations (SD), while categorical variables are presented as percentages. We estimated crude incidence rates of study outcomes per 1000 person-years. Cox proportional hazards models were used to estimate the association between ChEI use and clinical outcomes, calculating hazard ratios (HRs) with 95% confidence intervals (CIs). Time since the index date was used as the underlying timescale. Since there is a difference in memantine use between the ChEI treatment and non-use groups, we have further adjusted for memantine in the Cox model.

We also investigated the consistency of the effects of different types of ChEIs (donepezil, galantamine, or rivastigmine) vs. no ChEI on the outcomes.

Study covariates had no missing data except for baseline MMSE which was missing in 2.5 and 3.5% of PS matched cohort and total cohort, respectively. Missing MMSE was grouped into a ‘MMSE not recorded’ category.

All analyses were performed using R 3.4.3 software (The R Project for Statistical Computing, Vienna, Austria) and Stata version 17.0 (Stata Corp, College Station, TX).

## Results

*Figure 1* shows the flowchart of the patient selection. After exclusion criteria were applied, 1469 patients with HF and AD were eligible, of whom 809 were initiated on ChEI therapy within 90 days, whereas 660 were not. The majority of patients (89%) started ChEI treatment within the first 30 days after the dementia diagnosis. Using PS matching, two groups of patients with HF and AD with similar age, gender, and comorbidities were obtained, where one group started treatment with ChEI, and the other did not (*Figure 1*).

### Patient characteristics

Baseline characteristics of the cohort are shown, prior to (*Table 1*) and after (*Table 2*) PS matching.

Before PS matching, the total cohort consisted of 809 users of ChEI and 660 non-users. They differed significantly in several aspects, see *Table 1*. The non-users were older (84.2 vs. 81.5 years, *P* < 0.001) and had a lower MMSE score at baseline (19.4 vs. 21.5 points, *P* < 0.001). The non-users more often lived in a nursing home (14.8% vs. 6.3%, *P* < 0.001) and more often had CKD (12.6% vs. 6.7%, *P* < 0.001), history of stroke (11.7% vs. 8.3%, *P* < 0.030) atrial fibrillation (57.6% vs. 51.7%, *P* < 0.024), and fractures (35% vs. 28.6%, *P* < 0.008). The non-users were less often treated with statins (38.5% vs. 50.4%, *P* < 0.001) and ACEI/ARB (67.3% vs. 73.5%, *P* < 0.009), and more often with anxiolytics (24.2% vs. 17.9%, *P* < 0.003), antipsychotics (8.0% vs. 4.6%, *P* < 0.006) and memantine (39.8% vs. 5.2%, *P* < 0.001).

After PS matching, both groups were well-balanced with no significant differences in age, gender, MMSE, comorbidities, and medications except for dementia medication use (ChEIs and memantine, *Table 2*): Patients who were non-users of ChEI had more often memantine (42.4%) than the ChEI-treated group (5.9%).

In the propensity matched cohort, donepezil treated patients were less often diagnosed in specialist settings in comparison with patients treated with rivastigmine or galantamine. Moreover, memantine treatment in the ChEI groups within the first 3 months was low but more prevalent in those on rivastigmine (9,1%) and least used in the galantamine group (2.6%), see Supplementary material online, *Table S5*. Corresponding data for the whole cohort is found in the Supplementary material online, *Table S6*.

### Use of ChEI and outcome of hospitalizations for cardiovascular events or death

The PS matched cohort showed an association with a significant decrease (35%) in the risk of hospitalization due to composite cardiovascular diseases (CVD) events (HR: 0.65, 95% CI: 0.49, 0.87). When analysing the CVD separately, a significant (47%) decrease in risk of hospitalization due to HF (HR: 0.53, 95% CI: 0.38, 0.75) but not to stroke nor MI, was found (*Table 3*).

**Table 3:** Number of events, incidence rates, and adjusted hazard ratios for the association between ChEI initiation and deaths or hospitalizations for cardiovascular events in the propensity score matched cohort

	Number of patients	Events	Incidence rate per 1000 py^a^	HR^b^	(95% CI)
Deaths					
No ChEI	455	276	238.61	Ref	
Any ChEI	455	273	210.94	0.79*	0.66, 0.96
Hospitalization due to composite CVD events					
No ChEI	455	126	141.35	Ref	
Any ChEI	455	99	95.25	0.65**	0.49, 0.87
Hospitalization due to HF					
No ChEI	455	92	99.25	Ref	
Any ChEI	455	63	58.46	0.53***	0.38, 0.75
Hospitalization due to stroke					
No ChEI	455	24	23.52	Ref	
Any ChEI	455	24	20.77	0.92	0.48, 1.73
Hospitalization due to MI					
No ChEI	455	27	26.50	Ref	
Any ChEI	455	19	16.31	0.68	0.36, 1.31

*P < 0.05, **P < 0.01, ***P < 0.001.

a Incidence rates are presented as number of events per 1000 patient-years in PS matched cohort.

b Hazard ratio is obtained in PS matched cohort adjusting for the variables listed in *Table 1* and further adjusted for memantine.

**Figure 2:**
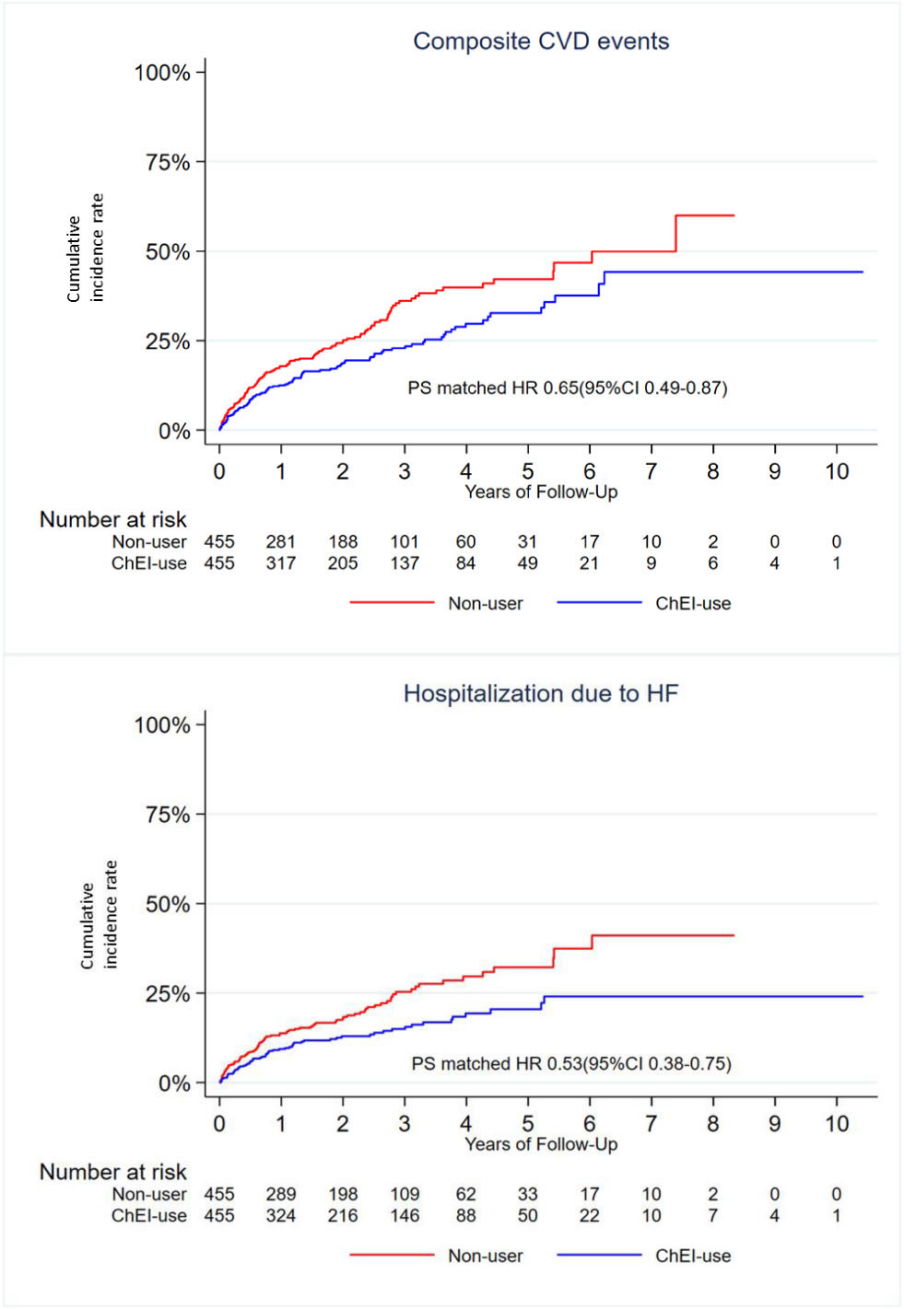
Patients treated with Cholinesterase inhibitors had lower risks of composite CVD events and lower risk of hospitalization due to HF compared to non-users.

*Figure 2* shows Kaplan–Meier curves of cumulative events of significantly decreased probability of composite CVD events and of probability of hospitalization with HF.

The PS matched cohort showed an association with a significant decrease of 21% in all-cause death for patients treated with ChEIs (HR: 0.79; 95% CI: 0.66, 0.96) compared with those without ChEI (*Table 3*).

**Figure 3:**
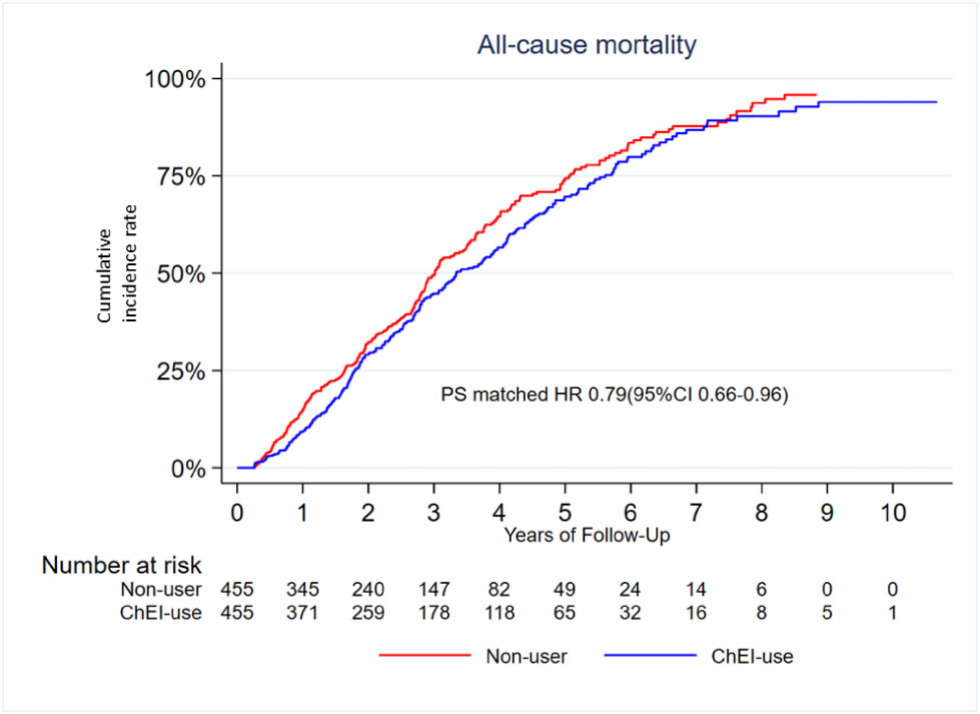
Patients treated with Cholinesterase inhibitors had lower mortality compared to non-users.

*Figure 3* shows Kaplan–Meier curves of cumulative events of significantly decreased mortality risk in the propensity matched cohort.

The results from the whole cohort are shown in Supplementary material online, *Table S3* and *Figures S1* and *S2*.

### Type of ChEI and risk of hospitalizations for cardiovascular events or death

*Table 4* shows the risk of hospitalizations for cardiovascular events in the PS matched score in relation to type of ChEI. A 41% decreased risk of hospitalization due to CVD (0.59, 95% CI: 0.42, 0.82, *P* < 0.01) and a 52% reduced risk of hospitalization due to HF (HR: 0.48, 95% CI: 0.32, 0.72, *P* < 0.001), was associated with donepezil treatment but not for galantamine nor rivastigmine.

**Table 4:** Number of events, incidence rates, and adjusted hazard ratios for the association between type of ChEIs and deaths or hospitalizations for cardiovascular events in the propensity score matched cohort

	Number of patients	Events	Incidence rate per 1000 py^a^	HR^b^	(95% CI)
All-cause death					
Non-use	455	276	238.61	Ref	
Donepezil	289	156	204.28	0.80*	0.64, 0.98
Rivastigmine	88	65	256.99	0.96	0.73, 1.28
Galantamine	78	52	187.32	0.64**	0.47, 0.87
Hospitalization due to composite CVD events					
Non-use	455	126	141.35	Ref	
Donepezil	289	55	87.06	0.59**	0.42, 0.82
Rivastigmine	88	22	113.36	0.76	0.48, 1.22
Galantamine	78	22	103.02	0.74	0.46, 1.18
Hospitalization due to HF					
Non-use	455	92	99.25	Ref	
Donepezil	289	35	53.96	0.48***	0.32, 0.72
Rivastigmine	88	14	67.09	0.61	0.34, 1.08
Galantamine	78	14	63.52	0.62	0.35, 1.10
Hospitalization due to stroke					
Non-use	455	24	23.52	Ref	
Donepezil	289	11	16.08	0.71	0.33, 1.53
Rivastigmine	88	8	36.52	1.60	0.68, 3.76
Galantamine	78	5	19.81	0.87	0.32, 2.42
Hospitalization due to MI					
Non-use	455	27	26.50	Ref	
Donepezil	289	11	16.05	0.65	0.31, 1.39
Rivastigmine	88	2	8.78	0.37	0.086, 1.61
Galantamine	78	6	23.88	1.10	0.43, 2.82

*P < 0.05, **P < 0.01, ***P < 0.001.

a Incidence rates are presented as number of events per 1000 patient-years in PS matched cohort.

b Hazard ratio is obtained in PS matched cohort adjusting for the variables listed in *Table 1* and further adjusted for memantine.

A decrease in all-cause mortality was significantly associated with donepezil (HR: 0.80, 95% CI: 0.64, 0.98) and galantamine (HR: 0.64, 95% CI: 0.47, 0.87), but not with rivastigmine.

The results for the whole cohort are shown in Supplementary material online, *Table S4*.

### Use of ChEI and hospitalizations for ChEI related severe side effects

ChEIs were not associated with significant differences in hospitalizations for severe side effects of bradycardia, AV block, or need for pacemaker implantation in the PS matched cohort (Supplementary material online, *Table S5A*) or in the total cohort (Supplementary material online, *Table S5B*).

## Discussion

Our main results are the following:

ChEIs were associated with decreased risk of all-cause death and with decreased risk of hospitalization due to composite CVD events. ChEIs were associated with decreased risk of hospitalization due to HF, but not due to only MI or only stroke. There was no significant difference in hospitalization for bradycardia, AV block, or implantation of the pacemaker between ChEI use and non-use.

When comparing the different ChEIs, we found that donepezil and galantamine but not rivastigmine were associated with a lower risk of death compared with non-users. We also found that donepezil and galantamine, but not rivastigmine were associated with lower risk of composite CVD events compared with non-users. Finally, donepezil was associated with a lower risk of hospitalization due to HF compared with non-users.

Our results, showing associations with a 21% decrease in all-cause death in AD patients, are in line with previous findings from our group^21,20,23,30^ and others (Hsieh *et al.*^6^). The positive effect on mortality among patients treated with ChEI is plausible, considering earlier reported enhanced effects on ACh signalling in the heart by ChEI, thereby reducing inflammation and reducing the sympathetic influence in the heart.^9^ Also direct effects of ChEIs on cardiomyocytes through increased parasympathetic activity with reduced heart rate, increased heart rate variability, and lowered myocardial oxygen demand, as well as indirect effects through anti-inflammatory pathways involving the modulation of nitric oxide signalling, regulation of redox states, improvement in mitochondrial function, calcium regulation, and protection against hypoxia-induced apoptosis and endothelial dysfunction have been reported.^10,31,32^ Moreover, the cholinergic alpha-7-nicotinic receptor (α7-nAChR) has been shown to be involved in reducing inflammatory neurotoxicity in stroke, AMI, sepsis, and AD.^33,34^ Li *et al.*^35^ reported that peripheral blockade of the α7-nAChR significantly increased the cardioprotective effects of donepezil in HF rats, whereas central blockade did not, suggesting that the peripheral activation of the α7-nAChR plays an important role in cholinergic pharmacotherapy for HF. Further, Vang *et al.*^36^ showed that α7-nAChR mediates ventricular fibrosis and diastolic dysfunction, relevant for HF.

Nordstrom *et al.*^21^ showed a decrease in all-cause death of a similar range as in our study among AD patients treated with ChEI, but also a decrease in risk of myocardial infarction. The latter was not found in this study. A reason for this discrepancy could be that our cohort included only patients with both HF and AD, while the Nordström study included AD patients irrespective of cardiovascular comorbidities.

If ChEI has disease-modulating effects on HF, a reduction in risk of hospitalization in this group would be expected. There was no reduction in hospitalization for MI or stroke, which makes it plausible that the reduction in risk of hospitalization due to HF was the main driver behind the reduced risk for hospitalization due to composite CVD events. Due to the character of HF, where patients are often recurrently hospitalized during deterioration of their HF, the risk of hospitalization due to HF is likely to be higher than the risk of other CVD events. Further, elderly patients with HF often have HFpEF,^2^ which is characterized by other aetiologies than myocardial infarction. HFpEF is more associated with inflammation which could strengthen a possible effect of ChEIs on hospitalization due to HF.

We found that galantamine had the strongest association with mortality reduction among the ChEIs (36%). Galantamine is the only ChEI which acts as an allosteric nicotinic modulator.^37^ Its dual effect as a modulator of α7-nAChR and an acetylcholinesterase inhibitor may at least partially explain this finding.

Our findings that reduction of all-cause death was associated with donepezil and galantamine, and that the risk for hospitalization due to HF was associated with donepezil, but not with rivastigmine need to be discussed. Rivastigmine has previously been reported to have a higher risk of mortality compared with donepezil.^38^

In our dataset, 86% of patients were using the rivastigmine patch, so the difference between rivastigmine, galantamine, and donepezil on the outcomes cannot be explained by the shorter half-life of rivastigmine.^39^ The difference may be attributed to several factors: (i) Molecular structure: Rivastigmine differs from donepezil and galantamine in that it inhibits both AChE and BuChE, while the others only inhibit AChE.^40^ This broader activity may affect the drug's impact on cardiovascular outcomes. (ii) Tissue selectivity: Rivastigmine has greater selectivity for the central nervous system over peripheral tissues, including the heart, which might explain its weaker effects on cardiovascular outcomes compared with donepezil and galantamine.^19^ (iii) Metabolism differences: Donepezil and galantamine are primarily metabolized by cytochrome P450 in the liver, whereas rivastigmine is metabolized by esterases.^41^ The cytochrome P450 pathway may lead to more active cardiovascular effects, potentially explaining the superior cardiovascular outcomes with donepezil and galantamine. (iv) Patient characteristics: Rivastigmine may be prescribed to more frail or cognitively impaired patients where the patch administration may be preferred over a pill, which may contribute to its association with worse outcomes. Our data show that rivastigmine was more commonly used in patients with lower MMSE scores and a higher use of antipsychotics, indicating more advanced Alzheimer's disease. These factors combined likely explain why rivastigmine shows different outcomes compared with donepezil and galantamine in terms of mortality and cardiovascular events. Information on frailty is, however, not available in our cohort.

When showing that ChEI-users had a better outcome, in terms of lower all-cause death and lower risk of hospitalization due to HF, it is of interest to discuss possible reasons for physicians to refrain from treatment with ChEIs, as our results suggest. Baseline characteristics prior to PS matching showed that non-users were older, had lower MMSE results, more often CKD and more fractures, atrial fibrillation, and strokes. The fear of cardiovascular side effects by the ChEIs may be one reason why the physicians refrain from prescribing ChEIs to this group. Increased rates of syncope and bradycardia in the presence of ChEI use have been reported by Hernandez.^29^ However, in the present study, there were no significant associations between ChEIs and the risk of bradycardia, AV block, or pacemaker implantation. A recent meta-analysis which did not find any significant associations between ChEI treatment and hospitalization due to bradycardia-induced events^22^ support our findings.

Non-ChEI users were also to a higher extent treated with antipsychotics and anxiolytics. The almost double use of antipsychotics suggests a presence of neuropsychiatric symptoms. Memantine is also reported to have a calming and small sedative effect, which may explain the higher use of memantine in this group.

Our data showed that memantine use was lower in the ChEI group compared with the non-user group, which prompted us to further adjust for memantine in our analysis. Additionally, previous studies have shown that memantine can have a beneficial effect on mortality,^42^ further supporting the advantage of ChEIs in clinical outcomes in our study.

### Strengths and limitations

One strength is the use of real-world clinical data in a cohort of HF with AD, treated or not with different ChEIs and the long follow-up. In addition to previous data on the positive effect of ChEIs on all-cause death for AD patients with risk for cardiovascular events, we have here also shown a similar association with reduced risk of mortality and of worsening of HF using the proxy of hospitalizations of HF in patients with both chronic HF and AD.

A limitation of this study is that, although we used PS matching to account for confounding by indication, we were only able to adjust for observed covariates and not for unmeasured or latent characteristics, which may be prevalent in the study population. In addition, before PS matching, the ChEI non-user group appeared to be in worse clinical condition, with a higher prevalence of CKD, stroke, and more patients living in nursing homes. These factors may have influenced the decision not to treat with ChEIs. Another limitation is that no laboratory samples in this cohort were collected and thus no information on inflammatory and other biomarkers is available.

Also, the patients were considered exposed throughout the whole follow-up period based on treatment status at study entry, according to the intention-to-treat design. We should note that despite the recommendation of chronic use, some patients may have discontinued treatment over an extended period. Consequently, we opted for this approach considering that the effects of ChEI on negative clinical outcomes are not instantaneous. Another limitation is the possibility that the attitudes towards ChEIs may have changed over the investigated years. During the first years of the studied period, the hesitation to use ChEIs due to the risk of bradycardia may have been more pronounced than later. However, ChEIs were not associated with adverse outcomes of bradycardia, AV block or pacemaker implantation in our analysis. Finally, lack of information on ejection fraction makes it not possible to relate our findings to the type of HF.

### Conclusions

This study supports the hypothesis that treatment with ChEIs is associated with a reduced risk of hospitalization due to HF as well as reduced mortality risk in patients with HF and AD, strengthening the recommendations for the use of ChEI in patients with HF and AD. However, among the ChEIs, varying patterns were observed, suggesting that further studies of different ChEIs are needed.

## Supplementary Material

pvae091_Supplemental_Files

## Data Availability

The data underlying this article will be shared on reasonable request to the corresponding author.
