# Supplementary material for: Cholinesterase inhibitors and reduced risk of hospitalization and mortality in patients with Alzheimer's dementia and heart failure
Source: Eur Heart J Cardiovasc Pharmacother. 2025 Jan 7;11(1):22–33. doi: 10.1093/ehjcvp/pvae091 (PMC11805694; doi:10.1093/ehjcvp/pvae091)
Supplement: pvae091_Supplemental_Files [file pvae091_supplemental_files.zip › Supplementary table 1.pdf]

Supplementary Table 1. Baseline characteristics, comorbidities, and medication

**Baseline characteristics**	**Definition**	
Age	Measured at index date	
Sex	Male/female	
MMSE score	Measured at dementia diagnosis date	
Memory clinics visit	Where the dementia diagnosis was issued	
Living situation	Living alone or with partner	
Nursing home care	Living in nursing home	
Dementia basic workups	Clock test, blood test, MMSE test, CT/MRI, Functional therapy, EEG test, Physiotherapy, SPECT	
**Comorbidities**	**ICD-10 codes**	
CCI	Calculated according to Charlson's index based on comorbidities	
Alcohol abuse	E244,F10,G312,G621,G721,I426,K292,K70,K860,O354, P043,Q860,T51,Y90,Y91,Z502,Z714	
Atrial Fibrillation	I48	
Cerebrovascular disease	G45-46, H340, I60-69	
Chronic kidney disease	I120, I131, N032-N0377, N052-N057, N18, N19, N250, T856, T857, Z490-Z492, Z940, Z992	
Chronic pulmonary disease	I278, I279, J40-47, J60-67, J684, J701, J703	
Congestive heart failure	I099, I110, I130, I132, I255, I420, I425-429, I43, I50, P290	
Depression	F32, F33, F204	
Diabetes	E10-14	
Fractures	S12, S22, S32, S42, S52, S62, S72, S82, S92, M90	
Hearing loss	H90, H91, H833	
Hypertension	I10-15	
Liver disease	K70-K77	
Myocardial infarction	I21, I22, I252	
Peptic ulcer disease	K25-K28	
Peripheral vascular disease	I70, I71, I731, I738, I739, I771, I790, I792, K551, K558, K559, Z958, Z959	
Rheumatic disease	M05, M06, M315, M32, M33, M34, M351, M353, M360	
Stroke	H341, I60, I61, I63, I64, I69	
**Medication**		**ATC codes**
ACEI/ARBs		C09
Acetylsalicylic acid		B01AC06, N02BA01
Antipsychotics		N05A
Antidepressants		N06A
Antithrombotics		B01
Anxiolytics		N05B
Beta blocking agents		C09
Calcium channel blockers		C08
ChEI use		
Donepezil		N06DA02

Galantamine		N06DA04
Rivastigmine		N06DA03
Diuretics		C03
Aldosterone antagonists		C03DA
Hypnotics		N05C
Memantine		N06DX01
NSAIDs		M01A
Statins		C10
Calendar year	Measured at diagnosis date	

Abbreviations: MMSE, Mini-Mental State Examination; ACEI, angiotensin-converting enzyme inhibitors; ARB, angiotensin receptor blockers; NSAIDs, nonsteroidal anti-inflammatory drugs.
