# Supplementary material for: Cholinesterase inhibitors and reduced risk of hospitalization and mortality in patients with Alzheimer's dementia and heart failure
Source: Eur Heart J Cardiovasc Pharmacother. 2025 Jan 7;11(1):22–33. doi: 10.1093/ehjcvp/pvae091 (PMC11805694; doi:10.1093/ehjcvp/pvae091)
Supplement: pvae091_Supplemental_Files [file pvae091_supplemental_files.zip › Supplementary table 2.pdf]

**Supplementary Table 2.** Definition of study outcomes

| Outcomes | ICD-10 codes |
|---|---|
| Deaths | |
| Composite CVD events | I099, I110, I130, I132, I255, I420, I425-429, I43, I50, P290<br>I21, I22, I252<br>H341, I60, I61, I63, I64, I69 |
| Congestive heart failure | I099, I110, I130, I132, I255, I420, I425-429, I43, I50, P290 |
| Myocardial infarction | I21, I22, I252 |
| Stroke | H341, I60, I61, I63, I64, I69 |
| Hospitalizations for cardiovascular side effects | |
| AV block | I440-443 |
| Bradycardia | R001 |
| Pacemaker implantation | Z950 |
