# Supplementary material for: Cholinesterase inhibitors and reduced risk of hospitalization and mortality in patients with Alzheimer's dementia and heart failure
Source: Eur Heart J Cardiovasc Pharmacother. 2025 Jan 7;11(1):22–33. doi: 10.1093/ehjcvp/pvae091 (PMC11805694; doi:10.1093/ehjcvp/pvae091)
Supplement: pvae091_Supplemental_Files [file pvae091_supplemental_files.zip › Supplementary table 3.docx]

#### **Supplementary Table 3**. Number of events, incidence rates, and adjusted hazard ratios for the association between ChEI initiation and deaths or hospitalizations for cardiovascular events in the whole cohort.

|  | **Number of patients** | **Events** | **Incidence rate per 1000 py^1^** | **HR^2^** | **(95%CI)** |
| --- | --- | --- | --- | --- | --- |
| **All-cause death** |  |  |  |  |  |
| No ChEI | 660 | 419 | 267.54 | Ref |  |
| Any ChEI | 809 | 465 | 181.90 | 0.78** | 0.66,0.91 |
| **Hospitalization due to composite CVD events** |  |  |  |  |  |
| No ChEI | 660 | 170 | 139.40 | Ref |  |
| Any ChEI | 809 | 199 | 97.36 | 0.68** | 0.53,0.87 |
| **Hospitalization due to HF** |  |  |  |  |  |
| No ChEI | 660 | 121 | 95.68 | Ref |  |
| Any ChEI | 809 | 131 | 61.48 | 0.61*** | 0.46,0.82 |
| **Hospitalization due to stroke** |  |  |  |  |  |
| No ChEI | 660 | 34 | 24.74 | Ref |  |
| Any ChEI | 809 | 51 | 22.19 | 0.87 | 0.51,1.49 |
| **Hospitalization due to MI** |  |  |  |  |  |
| No ChEI | 660 | 35 | 25.48 | Ref |  |
| Any ChEI | 809 | 36 | 15.60 | 0.62 | 0.35,1.08 |

^1^Incidence rates are presented as number of events per 1000 patient-years prior to propensity score in whole cohort.

**^2^**Hazard ratio is obtained in whole cohort adjusting for the propensity score and memantine.

*p<0.05, ** p<0.01, *** p<0.001
