# Supplementary material for: Cholinesterase inhibitors and reduced risk of hospitalization and mortality in patients with Alzheimer's dementia and heart failure
Source: Eur Heart J Cardiovasc Pharmacother. 2025 Jan 7;11(1):22–33. doi: 10.1093/ehjcvp/pvae091 (PMC11805694; doi:10.1093/ehjcvp/pvae091)
Supplement: pvae091_Supplemental_Files [file pvae091_supplemental_files.zip › Supplementary table 4.docx]

#### **Supplementary Table 4.** Number of events, incidence rates, and adjusted hazard ratios for the association between type of ChEIs and deaths or hospitalizations for cardiovascular events matching in the whole cohort.

|  | **Number of patients** | **Events** | **Incidence rate per 1000 py^1^** | **HR^2^** | **(95%CI)** |
| --- | --- | --- | --- | --- | --- |
| **All-cause death** |  |  |  |  |  |
| Non-use | 660 | 419 | 267.54 | Ref |  |
| Donepezil | 494 | 263 | 183.92 | 0.79** | 0.66,0.94 |
| Rivastigmine | 166 | 105 | 192.24 | 0.84 | 0.67,1.07 |
| Galantamine | 149 | 97 | 167.21 | 0.69** | 0.54,0.88 |
| **Hospitalization due to Composite CVD events** |  |  |  |  |  |
| Non-use | 660 | 170 | 139.40 | Ref |  |
| Donepezil | 494 | 106 | 90.83 | 0.63*** | 0.48,0.83 |
| Rivastigmine | 166 | 50 | 120.93 | 0.83 | 0.59,1.18 |
| Galantamine | 149 | 43 | 92.81 | 0.68* | 0.47,0.98 |
| **Hospitalization due to HF** |  |  |  |  |  |
| Non-use | 660 | 121 | 95.68 | Ref |  |
| Donepezil | 494 | 69 | 56.96 | 0.56*** | 0.40,0.78 |
| Rivastigmine | 166 | 33 | 75.26 | 0.75 | 0.49,1.13 |
| Galantamine | 149 | 29 | 60.30 | 0.64* | 0.41,0.99 |
| **Hospitalization due to stroke** |  |  |  |  |  |
| Non-use | 660 | 34 | 24.74 | Ref |  |
| Donepezil | 494 | 25 | 19.52 | 0.78 | 0.43,1.40 |
| Rivastigmine | 166 | 16 | 32.95 | 1.27 | 0.65,2.49 |
| Galantamine | 149 | 10 | 18.79 | 0.74 | 0.33,1.62 |
| **Hospitalization due to MI** |  |  |  |  |  |
| Non-use | 660 | 35 | 25.48 | Ref |  |
| Donepezil | 494 | 17 | 13.18 | 0.53 | 0.27,1.01 |
| Rivastigmine | 166 | 7 | 14.35 | 0.57 | 0.24,1.36 |
| Galantamine | 149 | 12 | 22.64 | 0.97 | 0.46,2.05 |

^1^Incidence rates are presented as number of events per 1000 patient-years in PS matched cohort.

^2^Hazard ratio is obtained in total cohort adjusting for the propensity score and memantine.

*p<0.05, ** p<0.01, *** p<0.001
