# Supplementary material for: Cholinesterase inhibitors and reduced risk of hospitalization and mortality in patients with Alzheimer's dementia and heart failure
Source: Eur Heart J Cardiovasc Pharmacother. 2025 Jan 7;11(1):22–33. doi: 10.1093/ehjcvp/pvae091 (PMC11805694; doi:10.1093/ehjcvp/pvae091)
Supplement: pvae091_Supplemental_Files [file pvae091_supplemental_files.zip › Supplementary table 5.docx]

#### **Supplementary Table 5:** Baseline characteristics, comorbidities, and medications stratified by ChEI treatment status within 3 months after diagnosis in the propensity score matching HF-AD cohort.

| **Baseline characteristics** | **Non** | **Donepezil** | **Rivastigmine** | **Galantamine** | **p-value** |
| --- | --- | --- | --- | --- | --- |
| N | 455 | 289 | 88 | 78 |  |
| Age, mean (SD) | 83.2 (6.2) | 83.7 (5.2) | 82.2 (6.3) | 82.7 (6.8) | 0.20 |
| Female | 58.5% | 57.4% | 61.4% | 57.7% | 0.93 |
| MMSE baseline, mean (SD) | 20.5 (4.9) | 20.5 (4.1) | 20.4 (5.1) | 20.8 (4.7) | 0.93 |
| MMSE strata |  |  |  |  | 0.21 |
| 0-9 | 2.4% | 1.0% | 2.3% | 0.0% |  |
| 10-19 | 33.4% | 36.7% | 30.7% | 37.2% |  |
| 20-24 | 41.3% | 45.3% | 45.5% | 32.1% |  |
| ≥25 | 20.4% | 15.2% | 17.0% | 26.9% |  |
| MMSE not recorded/not done | 2.4% | 1.7% | 4.5% | 3.8% |  |
| Specialist | 57.8% | 43.6% | 75.0% | 87.2% | <0.001*** |
| Living alone | 49.9% | 52.6% | 48.9% | 46.2% | 0.74 |
| Nursing home | 10.1% | 8.3% | 9.1% | 12.8% | 0.65 |
| **Comorbidities** |  |  |  |  |  |
| CCI, mean (SD) | 3.9 (1.9) | 4.0 (2.0) | 3.6 (1.7) | 3.6 (1.6) | 0.20 |
| Alcohol abuse | 2.0% | 1.4% | 0.0% | 5.1% | 0.086 |
| Atrial fibrillation | 55.8% | 57.8% | 55.7% | 56.4% | 0.96 |
| Cancer | 43.7% | 46.0% | 42.0% | 41.0% | 0.82 |
| Cerebrovascular diseases | 16.5% | 21.1% | 14.8% | 10.3% | 0.10 |
| Chronic kidney disease | 7.7% | 10.0% | 5.7% | 7.7% | 0.53 |
| Chronical pulmonary disease | 21.3% | 20.8% | 19.3% | 21.8% | 0.97 |
| Depression | 9.2% | 9.7% | 10.2% | 11.5% | 0.93 |
| Diabetes | 24.6% | 23.5% | 28.4% | 20.5% | 0.67 |
| Fractures | 30.8% | 29.4% | 26.1% | 32.1% | 0.81 |
| Hearing loss | 13.8% | 15.2% | 6.8% | 12.8% | 0.24 |
| Hypertension | 68.1% | 73.4% | 64.8% | 69.2% | 0.34 |
| Liver disease | 1.8% | 1.4% | 1.1% | 1.3% | 0.96 |
| Myocardial Infarction | 31.0% | 34.6% | 25.0% | 29.5% | 0.36 |
| Obesity by icd | 3.5% | 3.1% | 6.8% | 0.0% | 0.11 |
| Peripheral vascular disease | 10.3% | 10.4% | 8.0% | 9.0% | 0.90 |
| Peptic ulcers disease | 6.2% | 6.2% | 5.7% | 7.7% | 0.95 |
| Rheumatic diseases | 7.9% | 8.3% | 6.8% | 6.4% | 0.93 |
| Stroke | 9.7% | 12.1% | 5.7% | 7.7% | 0.28 |
| **Medication** |  |  |  |  |  |
| ACEI/ARB | 69.5% | 68.9% | 73.9% | 67.9% | 0.82 |
| Acetylsalicylic acid | 48.1% | 48.8% | 51.1% | 51.3% | 0.93 |
| Antidepressants | 30.3% | 30.1% | 20.5% | 30.8% | 0.29 |
| Antipsychotics | 6.8% | 5.9% | 6.8% | 6.4% | 0.97 |
| Antithrombotic | 91.2% | 91.0% | 94.3% | 91.0% | 0.79 |
| Antixiolytics | 22.2% | 22.1% | 23.9% | 28.2% | 0.68 |
| Beta-blocker | 72.5% | 75.8% | 73.9% | 66.7% | 0.42 |
| Calcium channel blocker | 22.6% | 23.2% | 19.3% | 24.4% | 0.86 |
| Diuretics | 70.5% | 68.9% | 77.3% | 73.1% | 0.47 |
| Aldosterone | 18.9% | 19.7% | 20.5% | 11.5% | 0.39 |
| Hypnotics | 31.6% | 31.8% | 29.5% | 37.2% | 0.74 |
| Memantine use | 42,4% | 5,9% | 9,1% | 2,6% | <0.001*** |
| NSAID | 7.3% | 5.9% | 6.8% | 10.3% | 0.60 |
| Statins | 43.1% | 42.2% | 38.6% | 37.2% | 0.71 |

*p<0.05, ** p<0.01, *** p<0.001
