# Supplementary material for: Cholinesterase inhibitors and reduced risk of hospitalization and mortality in patients with Alzheimer's dementia and heart failure
Source: Eur Heart J Cardiovasc Pharmacother. 2025 Jan 7;11(1):22–33. doi: 10.1093/ehjcvp/pvae091 (PMC11805694; doi:10.1093/ehjcvp/pvae091)
Supplement: pvae091_Supplemental_Files [file pvae091_supplemental_files.zip › Supplementary table 6.docx]

**Supplementary Table 6:** Baseline characteristics, comorbidities, and medications stratified by ChEI treatment status within 3 months after diagnosis in total cohort.

| **Baseline characteristics** | **Non** | **Donepezil** | **Rivastigmine** | **Galantamine** | **p-value** |
| --- | --- | --- | --- | --- | --- |
| N | 660 | 494 | 166 | 149 |  |
| Age, mean (SD) | 84.2 (6.3) | 82.1 (5.9) | 80.0 (7.2) | 80.8 (7.1) | <0.001*** |
| Female | 60.3 % | 57.5% | 53.0% | 59.7% | 0.36 |
| MMSE baseline, mean (SD) | 19.4 (5.3) | 21.1 (4.1) | 22.0 (4.7) | 22.2 (4.6) | <0.001*** |
| MMSE strata |  |  |  |  | <0.001*** |
| 0-9 | 4.7% | 0.6% | 1.2% | 0.7% |  |
| 10-19 | 38.9% | 31.0% | 19.3% | 25.5% |  |
| 20-24 | 35.6% | 45.1% | 49.4% | 34.2% |  |
| ≥25 | 15.3% | 21.7% | 27.7% | 37.6% |  |
| MMSE not recorded/not done | 5.5% | 1.6% | 2.4% | 2.0% |  |
| Specialist | 58.0% | 42.3% | 72.9% | 85.2% | <0.001*** |
| Living alone | 49.4% | 50.2% | 40.4% | 47.7% | 0.16 |
| Nursing home | 14.8% | 6.1% | 6.6% | 6.7% | <0.001*** |
| CCI, mean (SD) | 4.0 (1.9) | 4.0 (1.9) | 3.9 (1.9) | 3.6 (1.6) | 0.12 |
| Alcohol abuse | 2.0% | 1.2% | 1.8% | 4.0% | 0.18 |
| Atrial fibrillation | 57.6% | 54.5% | 47.6% | 47.0% | 0.028* |
| Cancer | 45.6% | 44.5% | 45.2% | 36.9% | 0.28 |
| Cerebrovascular diseases | 18.6% | 17.6% | 14.5% | 12.8% | 0.27 |
| Chronic kidney disease by icd10 | 12.6% | 7.7% | 5.4% | 4.7% | <0.001*** |
| Chronical pulmonary disease | 19.5% | 20.9% | 19.3% | 18.8% | 0.92 |
| Depression | 9.7% | 8.1% | 7.8% | 7.4% | 0.67 |
| Diabetes | 25.3% | 25.7% | 30.7% | 22.8% | 0.41 |
| Fractures | 35.0% | 28.7% | 28.9% | 27.5% | 0.069 |
| Hearingloss | 14.5% | 14.6% | 8.4% | 10.1% | 0.099 |
| Hypertension | 70.0% | 71.9% | 66.9% | 64.4% | 0.29 |
| Liver disease | 1.7% | 1.2% | 1.2% | 0.7% | 0.78 |
| Myocardial Infarction | 31.8% | 32.6% | 28.9% | 31.5% | 0.85 |
| Obesity by icd | 3.5% | 3.6% | 5.4% | 2.0% | 0.44 |
| Peptic ulcers disease | 7.0% | 6.7% | 3.6% | 4.7% | 0.35 |
| Peripheral vascular disease | 9.1% | 12.8% | 11.4% | 11.4% | 0.25 |
| Rheumatic diseases | 8.6% | 7.5% | 6.6% | 4.0% | 0.26 |
| Stroke | 11.7% | 9.3% | 5.4% | 8.1% | 0.077 |
| **Medication** |  |  |  |  |  |
| ACEI/ARB | 67.3% | 74.1% | 76.5% | 68.5% | 0.023* |
| Acetylsalicylic acid | 47.4% | 49.4% | 49.4% | 53.7% | 0.57 |
| Antidepressants | 30.8% | 32.2% | 24.7% | 24.8% | 0.14 |
| Antipsychotics | 8.0% | 4.7% | 4.8% | 4.0% | 0.054 |
| Antithrombotic | 90.8% | 91.1% | 91.6% | 89.3% | 0.90 |
| Anxiolytics | 24.2% | 17.8% | 15.1% | 21.5% | 0.013* |
| Beta-blocker | 74.5% | 76.1% | 77.1% | 70.5% | 0.49 |
| Calcium channel blocker | 21.5% | 24.9% | 21.1% | 23.5% | 0.54 |
| Diuretics | 73.6% | 67.8% | 74.7% | 67.1% | 0.076 |
| Aldosterone | 17.4% | 20.6% | 24.7% | 13.4% | 0.037* |
| Hypnotics | 32.1% | 29.6% | 24.7% | 36.9% | 0.094 |
| Memantine | 39,8% | 5,1% | 7,8% | 2,7% | <0.001*** |
| NSAID | 6.2% | 6.3% | 10.8% | 10.1% | 0.080 |
| Statins | 38.5% | 50.6% | 51.2% | 49.0% | <0.001*** |

*p<0.05, ** p<0.01, *** p<0.001
