# Supplementary figures and images for: Cholinesterase inhibitors and reduced risk of hospitalization and mortality in patients with Alzheimer's dementia and heart failure
Source: Eur Heart J Cardiovasc Pharmacother. 2025 Jan 7;11(1):22–33. doi: 10.1093/ehjcvp/pvae091 (PMC11805694; doi:10.1093/ehjcvp/pvae091)

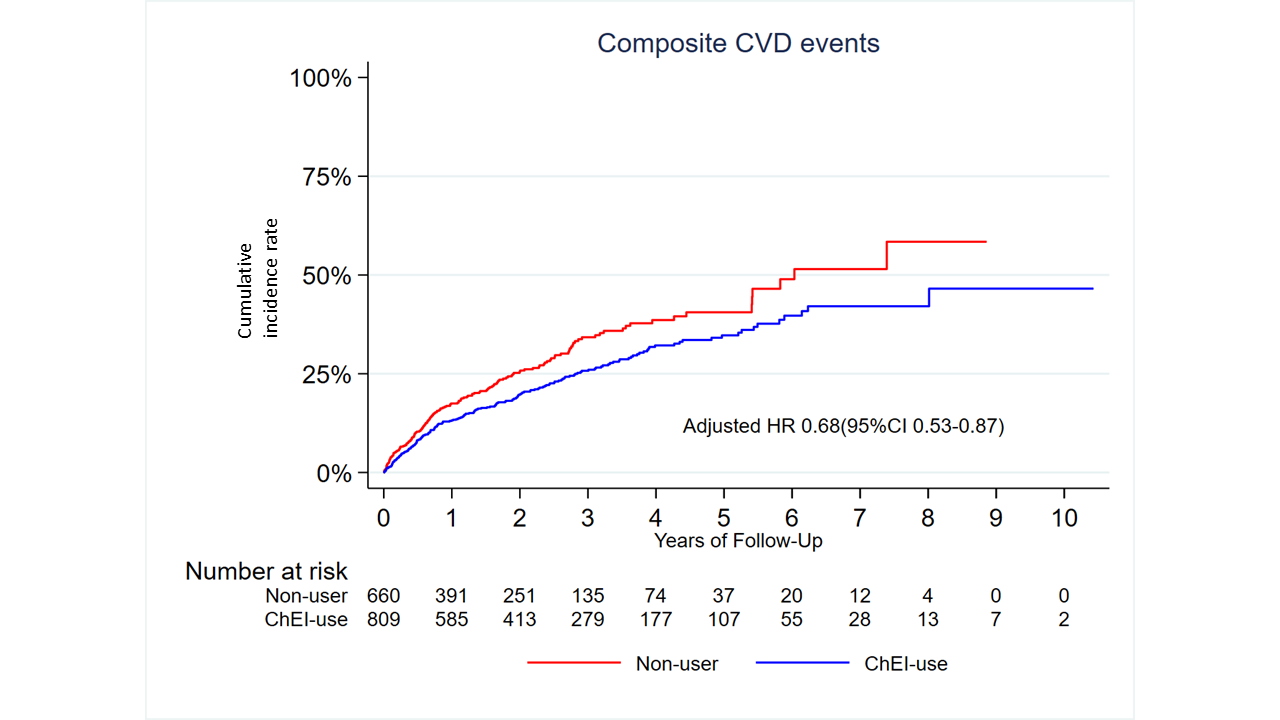


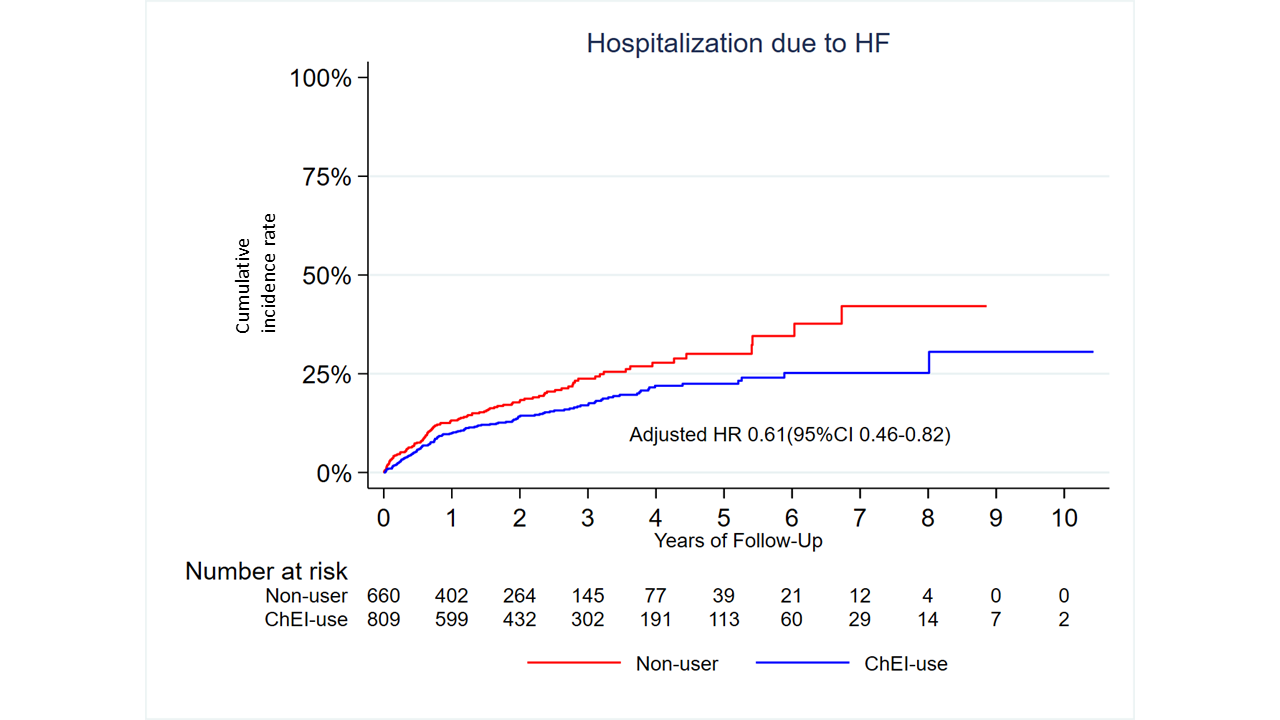

Supplement: pvae091_Supplemental_Files [file pvae091_supplemental_files.zip › Supplementary figure 1.docx]

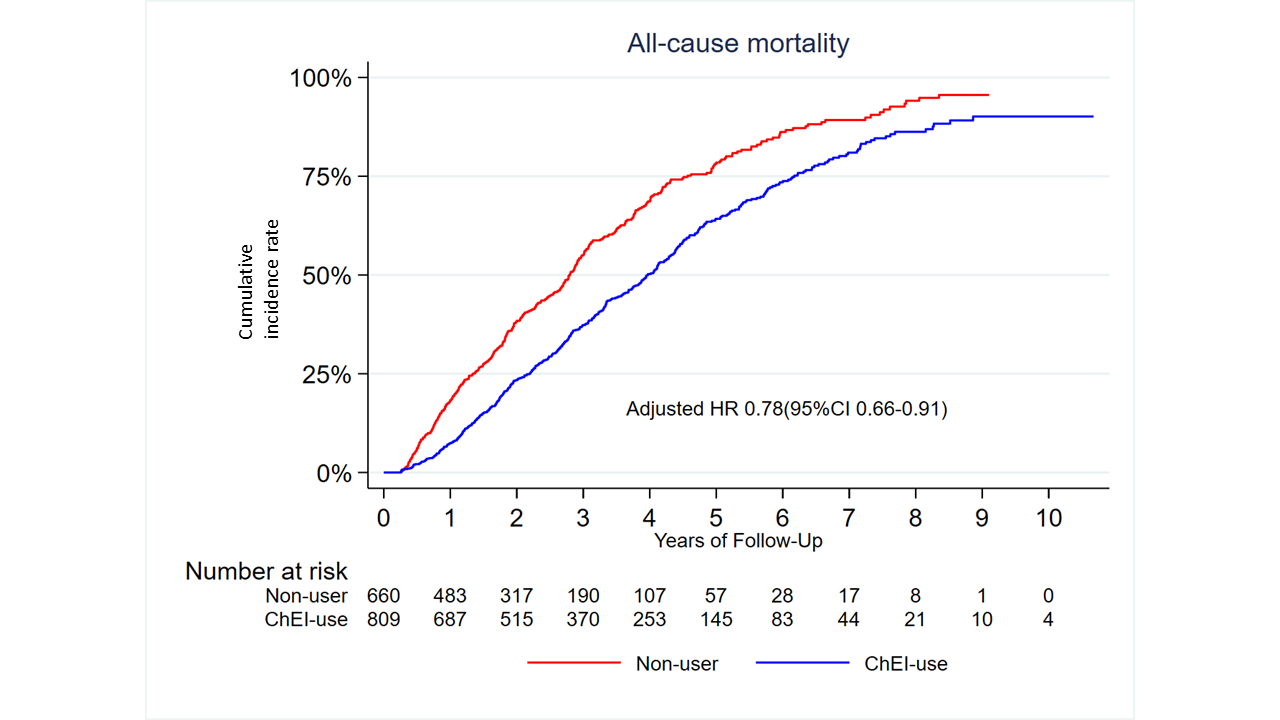

Supplement: pvae091_Supplemental_Files [file pvae091_supplemental_files.zip › Supplementary figure 2.docx]
